# A Fully Automatic Artificial Intelligence System Able to Detect and Characterize Prostate Cancer Using Multiparametric MRI: Multicenter and Multi-Scanner Validation

**DOI:** 10.3389/fonc.2021.718155

**Published:** 2021-10-01

**Authors:** Valentina Giannini, Simone Mazzetti, Arianna Defeudis, Giuseppe Stranieri, Marco Calandri, Enrico Bollito, Martino Bosco, Francesco Porpiglia, Matteo Manfredi, Agostino De Pascale, Andrea Veltri, Filippo Russo, Daniele Regge

**Affiliations:** ^1^ Department of Radiology, Candiolo Cancer Institute, FPO-IRCCS, Candiolo, Italy; ^2^ Department of Surgical Sciences, University of Turin, Turin, Italy; ^3^ Radiology Unit, Azienda Ospedaliera Universitaria (AOU) San Luigi Gonzaga, Orbassano, Italy; ^4^ Department of Oncology, University of Turin, Turin, Italy; ^5^ Department of Pathology, San Luigi Gonzaga Hospital, University of Turin, Orbassano, Italy; ^6^ Department of Pathology, San Lazzaro Hospital, Alba, Italy; ^7^ Department of Urology, San Luigi Gonzaga Hospital, University of Turin, Orbassano, Italy

**Keywords:** prostate cancer, artificial intelligence, automatic segmentation, aggressiveness score, external validation, magnetic resonance imaging

## Abstract

In the last years, the widespread use of the prostate-specific antigen (PSA) blood examination to triage patients who will enter the diagnostic/therapeutic path for prostate cancer (PCa) has almost halved PCa-specific mortality. As a counterpart, millions of men with clinically insignificant cancer not destined to cause death are treated, with no beneficial impact on overall survival. Therefore, there is a compelling need to develop tools that can help in stratifying patients according to their risk, to support physicians in the selection of the most appropriate treatment option for each individual patient. The aim of this study was to develop and validate on multivendor data a fully automated computer-aided diagnosis (CAD) system to detect and characterize PCas according to their aggressiveness. We propose a CAD system based on artificial intelligence algorithms that a) registers all images coming from different MRI sequences, b) provides candidates suspicious to be tumor, and c) provides an aggressiveness score of each candidate based on the results of a support vector machine classifier fed with radiomics features. The dataset was composed of 131 patients (149 tumors) from two different institutions that were divided in a training set, a narrow validation set, and an external validation set. The algorithm reached an area under the receiver operating characteristic (ROC) curve in distinguishing between low and high aggressive tumors of 0.96 and 0.81 on the training and validation sets, respectively. Moreover, when the output of the classifier was divided into three classes of risk, i.e., indolent, indeterminate, and aggressive, our method did not classify any aggressive tumor as indolent, meaning that, according to our score, all aggressive tumors would undergo treatment or further investigations. Our CAD performance is superior to that of previous studies and overcomes some of their limitations, such as the need to perform manual segmentation of the tumor or the fact that analysis is limited to single-center datasets. The results of this study are promising and could pave the way to a prediction tool for personalized decision making in patients harboring PCa.

## 1 Introduction

Prostate cancer (PCa) is the most common malignancy in men in both Europe and the United States (1, 2). Improved treatment and earlier diagnosis have almost halved PCa-specific mortality since the 1990s (3). However, after the introduction of prostate-specific antigen (PSA), millions of men with clinically insignificant cancer not destined to cause death have received treatment, with no beneficial impact on overall survival (3, 4). It is well understood that whole gland treatments could be avoided in men with indolent PCa, provided that they are properly selected (5, 6). Indeed, the ProtecT trial showed that in men with clinically localized PCa, active monitoring, radiotherapy, and prostatectomy have no statistically significant differences in cancer-specific mortality after 10 years of follow-up (7). However, trials designed in the PSA testing era used systematic biopsy to diagnose PCa, which is known to underestimate both PCa aggressiveness and extension  (8). The Gleason grade (GG) criteria, published in 2013, underline the importance of properly classifying PCa by correlating pathology to prognosis (9). The most significant classification change is the separation of patients classified with Gleason score (GS) 7 in two different categories: GG 2, with a GS of 3 + 4, including patients with a more favorable prognosis than GG 3 patients, with a GS of 4 + 3 (10). Notwithstanding, treatment decisions are still based on PSA, biopsy, and staging (11).

Since 2020, the European Association of Urology guidelines strongly recommend MRI prior to prostate biopsy to localize cancer and to diagnose extra-prostatic extension (11). MRI is superior to clinical staging, as it increases detection of PCa and allows a more precise risk classification (12–14). Moreover, men with suspicious findings at imaging can benefit from fusion biopsy, merging MRI information with real-time ultrasound (US), providing higher sampling precision and improved diagnostic yield (15, 16). Unfortunately, MRI of the prostate largely relies on qualitative assessment (17) and suffers from large inter-reader variability, being strongly related to readers’ expertise (18). Furthermore, qualitative assessment does not allow determination of tumor aggressiveness.

In recent years, efforts have been made to determine if quantitative radiomics signatures could allow better assessment of PCa aggressiveness, using both conventional statistics metrics (19–21) and higher-order texture features (13, 22–26) derived from T2-weighted (T2w) images and apparent diffusion coefficient (ADC) maps. Moreover, machine learning (ML) methods have been implemented to sift through the large amounts of high-dimensional data provided by radiomics, to optimize accuracy, reproducibility, and throughput (27–29). Unfortunately, most previous studies are not easily transferable to clinical practice either because they lack validation on external datasets or, most importantly, due to the absence of an automatic pipeline to segment and characterize tumor regions without human intervention (30).

The aim of this study was to develop and validate on multivendor data a fully automated computer-aided diagnosis (CAD) system based on artificial intelligence, to localize, segment, and classify PCa lesions according to their aggressiveness. The proposed tool aims at providing better stratification of men with suspicion of PCa, to support physicians in the selection of the most appropriate treatment option for each individual patient.

## 2 Materials and Methods

### 2.1 Patients

**Figure 1:**
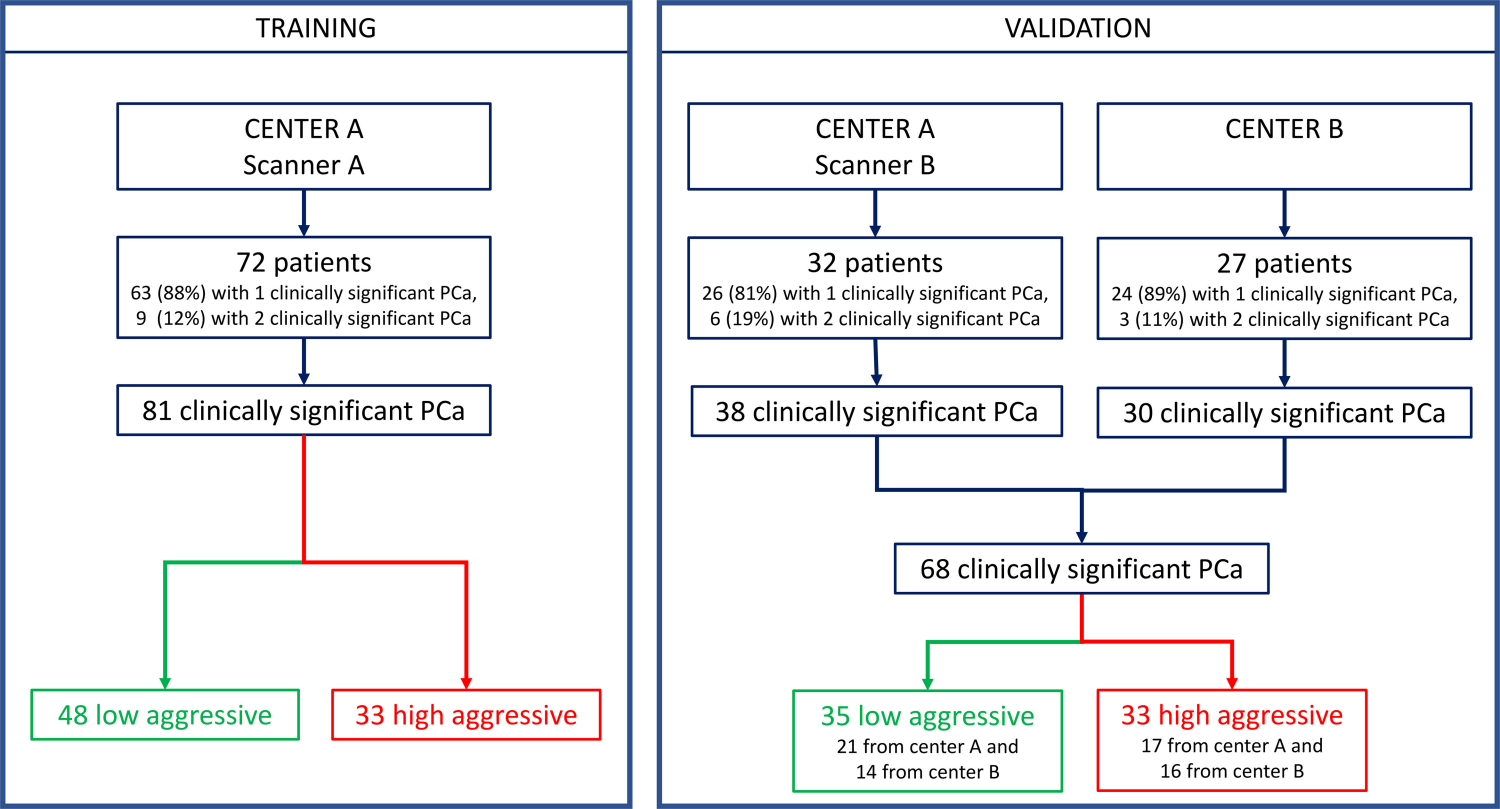
Composition of training and validation sets.

This multicenter retrospective study was approved by the local Ethics Committees. It was in accordance with the Declaration of Helsinki, and all participants signed informed consent forms. Inclusion criteria were the following: a) multiparametric (mp)-MRI examination performed between April 2010 and November 2019, including axial T2w, diffusion-weighted (DW), and dynamic contrast-enhanced (DCE)-MRI sequences; b) biopsy-proven PCa; c) radical prostatectomy (RP) within 3 months of mp-MRI; and d) a clinically significant peripheral zone lesion (tumor volume ≥0.5 ml, GS ≥ 6) (31) at the whole-mount histopathologic analysis. Exclusion criteria were a) low mp-MRI quality, b) patients in whom biopsy was performed less than 8 weeks after mp-MRI, and c) pathologically confirmed PCas that were not detected by the CAD system (18, 32). MRI scans collected at the Candiolo Cancer Institute (center A) were used for both training and validation, while MRI collected from the San Luigi Hospital (center B) were used only as the second holdout validation set (see flow chart in **Figure 1**).

### 2.2 MRI Acquisition

At center A, images were obtained either with a 1.5-T scanner (Signa Excite HD, GE Healthcare, Milwaukee, WI, USA) using a four-channel phased-array coil combined with an endorectal coil (Medrad, Indianola, PA, USA) or with a 1.5-T scanner (Optima MR450w, GE Healthcare, Milwaukee, WI, USA) using both 32-channel phased-array and endorectal coils (Medrad, Indianola, PA, USA). At center B, images were acquired with a 1.5-T scanner (Philips Achieva 1.5T DS, Nederland B.V., PC Best, Netherlands) using a five-channel phased-array coil combined with an endorectal coil (Medrad, Indianola, PA, USA). In both centers, the DCE sequence was triggered to start simultaneously with the power injection of 0.1 mmol/kg of gadobutrol (Gadovist, Bayer Schering, Berlin, Germany) through a peripheral line at 0.7 ml/s, followed by infusion of 20 cm^3^ of normal saline at the same rate. Axial T2w, DW, and DCE sequence parameters are detailed in **Supplementary Table 1**. The average time to complete the whole MRI examination, including two additional T2w scans in the sagittal and coronal planes and an additional DW sequence with a higher b-value, was 40 min. Imaging parameters satisfied the scanning European Society of Urogenital Radiology (ESUR) guidelines for prostate imaging (33).

### 2.3 Histopathologic Analysis and Reference Standard

Whole-mount histological sections resected from the RP specimens were used as the reference standard. After RP, surgical specimens were step-sectioned at 3-mm intervals perpendicular to the long axis (apical–basal) of the gland, with the same inclination as that of the axial T2w images (32, 34). The bases and the apexes were cut longitudinally. Then, 5-µm sections were taken from each thick slice and stained with hematoxylin and eosin. The same experienced pathologist (with 24 years of experience in pathology and 20 years attending uropathology) outlined each clinically significant tumor on microscopic slices and assigned a pathological GG (35, 36). Clinically significant PCa was defined as a tumor with volume >0.5 ml and/or pathological GS >= 6 (31). All malignant lesions were then contoured with a marker on the microscopic slice, and then each section was scanned for comparison with MRI findings.

### 2.4 Prostate Cancer Automatic Segmentation

**Figure 2:**
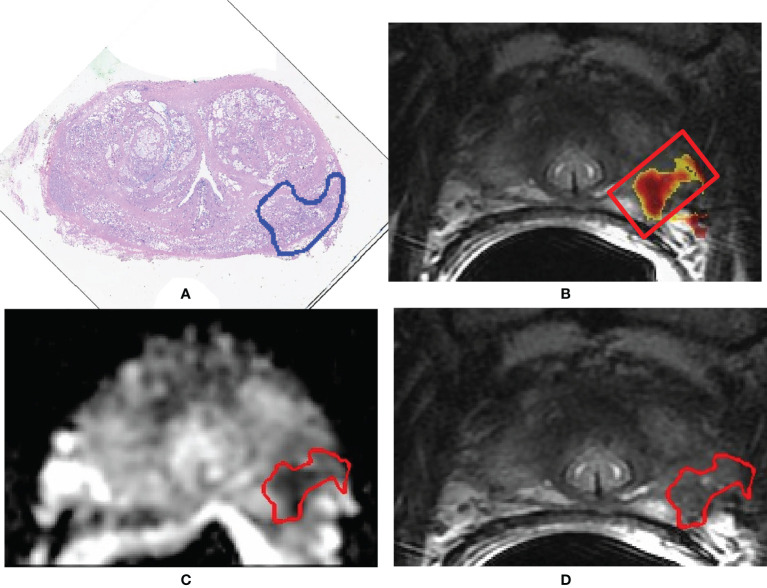
A 71-year-old man with Gleason score 3 + 4 (GG 2) prostate cancer contoured on the whole-mount prostatectomy slice **(A)**. Findings of the computer-aided diagnosis system were shown to the radiologist superimposed to the T2-weighted (T2w) image **(B)**. Using pathology results and MR images, the radiologist selected the region of interest containing the whole 3D tumor segmentation (red box in image **B**). Texture features were extracted from both the apparent diffusion coefficient (ADC) map and the T2w image from voxels belonging to the 3D segmentation of the tumors shown in red in **(C, D)**. **(C)** Example of a slice of the segmentation mask of the tumor superimposed to the ADC map; **(D)** example of a slice of the segmentation mask of the tumor superimposed to the T2w image.

Segmentation of the whole PCa was performed using a previously validated CAD system (18, 32, 37). The CAD system consists of multiple sequential steps, briefly reported in this paragraph. First, all MRI sequences are registered (37) to correctly compare voxels coming from different images. Once all datasets are aligned, quantitative features are extracted from each voxel, including ADC value, normalized T2w signal intensity, a_0_, and r of phenomenological universality (PUN) model (38) and fed into a support vector machine (SVM) classifier to produce a voxel-wise malignancy probability map. Finally, all voxels having probability to be malignant <60%, ADC values either <200 mm^2^/s or >1,600 mm^2^/s, and maximum contrast uptake in the first minute <100% are discarded. Only connected regions with area >100 mm^2^ are kept and considered as candidate to be PCa. This size represents 60% of the volume of the smallest clinically significant PCa, i.e., 0.5 ml (31). Once the automatic 3D lesion segmentation was provided by the CAD system, an experienced radiologist (>500 prostate mp-MRI studies interpreted per year for 10 years) selected the actual lesion by comparing MRI sequences with the outlines drawn by the pathologist on digital images of the pathologic slices (**Figure 2**). When pathological microslices and axial MR images were not perfectly overlapped, usually due to modified prostate shape soaked by formaldehyde, the radiologist and the pathologist established in consensus the locations of tumors with respect to identifiable anatomic landmarks (e.g., adenoma nodule, urethra, ejaculatory ducts, and benign prostatic hyperplasia).

### 2.5 Texture Analysis and Quantitative Features

To extract quantitative and texture features, we used an in-house, image biomarker standardization initiative (IBSI)-compliant software (39), implemented using C++ and the ITK libraries. The IBSI reference provides standardized image biomarker nomenclature and definitions, and a standardized general image processing workflow, allowing to overcome the lack of reproducibility and validation that can affect many radiomics studies (39).

First, we applied a de-noising step with a Gaussian filter (σ = 0.5 mm) (40). Then, we interpolated MR images and tumor masks to isotropic voxel spacing of 0.5 mm, using a trilinear interpolator for MR images (41) and the nearest neighbor interpolator for masks, since it produces meaningful masks. Interpolation is needed to produce 3D texture features that are rotationally invariant, and it allows comparison between image data from different samples (42). Once all images were interpolated, we re-segmented the masks of all PCas between the first and 99th percentiles of the region of interest (ROI) to remove outliers. Finally, we discretized MR images using a fixed number of bins (n=64). The fixed number of bin introduces a normalizing effect, which may be beneficial when intensity units are arbitrary (e.g., raw MRI data and many spatial filters), and where image contrast is considered important (42). Discretization of image intensities inside the ROI is often required to make calculation of texture features tractable (43). Details regarding image processing and feature extraction method according to the IBSI reporting guidelines are described in **Supplementary Table 2**.

Finally, we computed the following 92 features: 1) ROI volume (mm^3^); 2) eight intensity-based statistics from the ADC maps, i.e., mean, 25th percentile, 50th percentile, 75th percentile, skewness, kurtosis, intensity kurtosis, and intensity variance; 3) mean intensity histogram from the ADC maps; 4) 50 features derived from the gray-level co-occurrence matrices (GLCMs), 25 from the ADC maps, and 25 from the T2w images; 5) 32 features derived from the gray-level run length matrices (GLRLMs), 16 from the ADC maps, and 16 from the T2w images. Since the T2w image is not a quantitative map and consequently it suffers from high variability between scanners and acquisition protocols, we decided to compute only the texture-based features from this type of image and not to compute the intensity-based statistics.

The GLCM was computed at one pixel distance (Δx = 1), and both GLCM and GLRLM were computed using 32 bins, for each of the 13 directions of a 3D image, and then averaged to enable the method to be rotationally invariant to the distribution of texture, i.e., 3D merged features according to IBSI nomenclature.

A list of all features is provided in **Supplementary Table 2**.

### 2.6 Feature Selection and Classifier Development

Feature selection (FS) is a process of choosing a subset of original features to reduce dimensionality, remove irrelevant data, increase learning accuracy, and improve result comprehensibility (44, 45). The main idea of FS is to select an optimal subset of input variables, by removing features with little or no predictive information. In this study, FS was performed using a twofold approach: first, we discarded highly correlated features, and then we used a wrapper method to select the optimal subset of variables and avoid overfitting. We first normalized all features using the min–max scaling method to obtain the same range of values for each feature, i.e., 0–1; then, on the training set, we computed the bootstrapped area under the receiver operating characteristic (ROC) curve (AUC) for each feature and the correlation matrix between all features. When two features showed a correlation higher than 0.9, we removed the features having the lowest value of AUC. Once all highly correlated features were removed, we developed the FS wrapper method, in which a threshold on the number of features that would be included in the classification model was defined accounting for the performance of a classifier (46, 47). More specifically, n feature subsets were created by including the first n features ordered by AUC. Then each subset was fed into an SVM classifier that used a third-order polynomial function (box constraint = 1). To avoid overfitting, we used a k-fold cross-validation (CV) with k = 4. The k-fold CV consists in partitioning the dataset into k-fold and performing training on all but one fold and testing on the left-out fold. This procedure is repeated until each fold has been used. Performance of each of the n SVM classifier, i.e., trained with the nth subset, was measured as the mean accuracy of the k training sets and the accuracy obtained on the test set. Finally, we selected the n threshold, according to the point of overfitting, i.e., the point in which accuracy on the training set keeps increasing while accuracy on the test set starts decreasing.

Once the best feature subset was selected, we optimized the SVM *via* a grid search using incremental values of box constraint from 1 to 50 (step of 1) and the same k-fold CV (k = 4). FS and development of the classifier were performed using Matlab (v R2019a).

### 2.7 Statistical Analysis

The endpoint of this study was to evaluate the performance of the radiomics score in distinguishing between low and high aggressive tumors. Performances were evaluated by means of both monoparametric and multiparametric analyses. For the first analysis, the Mann–Whitney U test was used to compare each texture feature of both T2w images and ADC maps for differentiation of the two risk groups. Bootstrap AUCs, along with 95% confidence intervals, were computed for all features in the training set.

For the multiparametric analysis, we first computed the AUC obtained with the SVM for both training and validation sets. Then, we selected the most cost-effective cutoff (Youden’s index) of the ROC curve obtained in the training phase, and we applied the same cutoff to the validation datasets. Youden’s index is the point on the ROC curve that has the minimum distance to the upper left corner (where sensitivity = 1 and specificity = 1) and represents the value for which both sensitivity and specificity are maximized. We also evaluated results at the cutoff that maximizes the negative predictive value (NPV), which is a measure that indicates the real number of low aggressive tumors among all tumors that are classified as low aggressive. This metric is important from a clinically point of view, since, ideally, we do not want to misclassify as low aggressive any aggressive tumor and consequently not to treat it, even at the cost of overtreating a few of low aggressive tumors. Once the cutoffs were selected, we computed accuracy, sensitivity, specificity, NPV, and positive predictive value (PPV). Finally, we divided our radiomics score into three levels of suspicious of aggressiveness (Agg-score): a) indolent: not likely to be aggressive, if the radiomics score was from 0 to −15% of the best cutoff; b) indeterminate: patient needs further investigations with biopsy, when the radiomics score is comprised between −15% of the best cutoff and the best cutoff; and c) aggressive: likely to be aggressive, if the radiomics score was higher than the best cutoff.

AUCs of the most discriminant features and of the SVM were compared using Delong’s test (48). Results between performance obtained in the two validation centers were compared using the chi-squared test. A p-value of less than 0.05 was considered a statistically significant result.

## 3 Results

Patients’ demographics and clinical data are presented in **Table 1**. The training set included 72 patients for a total of 81 clinically significant peripheral PCas, while the multicenter validation set was composed of 59 patients for a total of 68 clinically significant peripheral PCas. Details of the dataset composition are described in **Figure 1**.

**Table 1:** Clinical and demographic characteristics of the patient cohort.

Number of patients (n)	Total	Training	Validation 1	Validation 2
	131	72	32	27
Number of clinically significant PCas (n)	149	81	38	30
Median age, years [IQR]	66 [61–70]	65 [60–70]	67 [60–70]	68 [63–72]
Median PSA ng/ml [IQR]	6.3 [5.3–9.1]	6.1 [4.9–8.1]	6.0 [5.5–11]	7.0 [5.2–10.3]
Pathologic stage at prostatectomy, n (%)				
pT2a	22 (17)	9 (12)	7 (22)	6 (22)
pT2c	48 (36)	33 (46)	10 (31)	5 (19)
pT3a	43 (33)	18 (25)	13 (41)	12(44)
pT3b	18 (14)	12 (17)	2 (6)	4(15)
Gleason score [Gleason grade], n (%)				
3+3 [GG 1]	8 (5)	4 (5)	1 (3)	3 (10)
3+4 [GG 2]	75 (51)	44 (54)	20 (53)	11 (37)
4+3 [GG 3]	42 (28)	19 (24)	10 (26)	12 (40)
4+4 or higher [GG 4–5]	24 (16)	14 (17)	7 (18)	4 (13)
Median largest diameter (mm) [IQR]	13 [10–18]	13 [10–18]	12 [7–16]	16 [12–20]

PSA, prostate-specific antigen; PCa, prostate cancer; IQR, interquartile range.

There were no statistically significant differences in PSA, age, and PCa largest diameter when comparing the validation set and the training set. Age, PSA, and PCa largest diameter were higher in the group of patients with aggressive PCa (p = 0.007, p = 0.0001, and p = 0.0001 respectively).

### 3.1 Monoparametric Analysis

**Figure 3:**
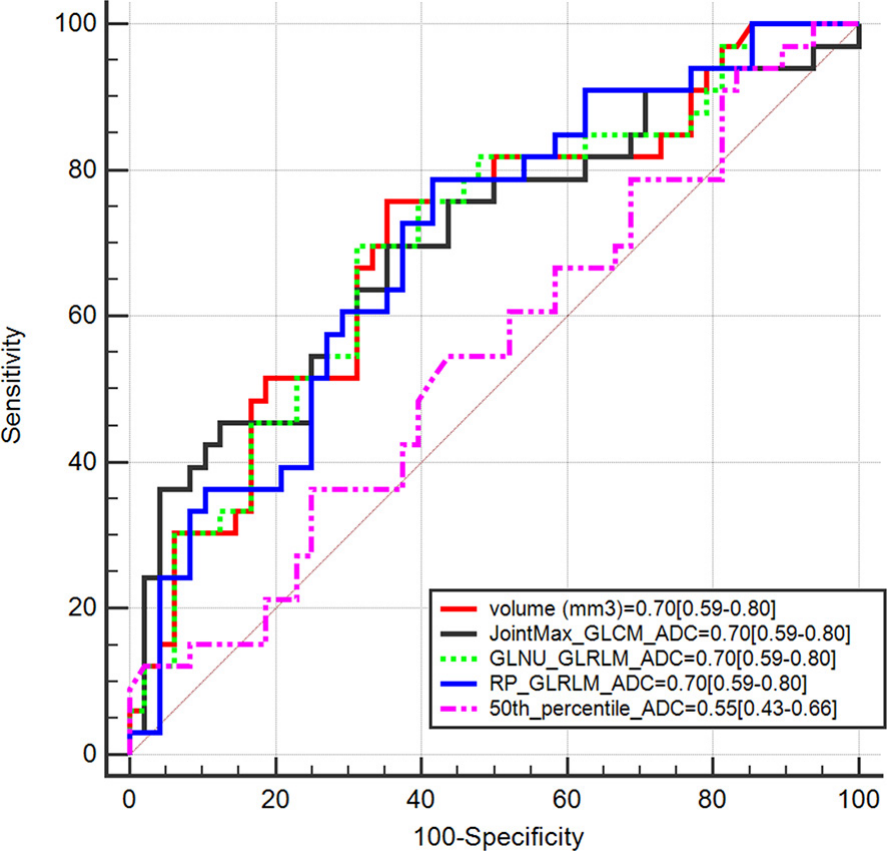
Area under the receiver operating characteristic curve (AUC) of radiomics features having AUC ≥ 0.7 compared with AUC of the 50th percentile of apparent diffusion coefficient (ADC).

The Mann–Whitney test showed that 56 out of 92 features were statistically different between GG ≤ 2 PCas and GG > 2 PCas in the training set. Among them, 55 had bootstrapped AUC statistically higher than 0.5. **Figure 3** compares features having AUC ≥ 0.7 with the AUC of the 50th percentile of the ADC (0.55), which is a parameter that radiologists usually considered to assess PCa aggressiveness.

**Table 2** shows performances of features having AUC ≥ 0.7. Sensitivity, specificity, PPV, and NPV were computed on both the training and validation sets at Youden’s index obtained on the training set. The best performance was reached using the lesion volume, with sensitivity of 76% and specificity of 65% in the training set. However, most of these features did not show good performances when the same cutoff was applied on the validation set, and either sensitivity or specificity dropped significantly. This is probably due to the fact that individual features are not largely different in the low and aggressive tumors; therefore a cutoff found on a dataset is not generalizable to another one, i.e., the validation set. Only, RP_GLRLM_ADC showed similar performance between the training and validation sets; however, results obtained on both datasets are not high enough to develop a radiomics score based only on this feature. Moreover, sensitivity of RP_GLRLM_ADC was significantly different between center A and center B, 41% and 87% respectively, meaning that its performance depends on the MR scanner. All the other metrics were not different between centers A and B.

**Table 2:** Performance of monoparametric analysis on both training and validation sets for radiomics features having AUC >= 0.7.

		Training set				Validation set			
Feature	Best thresh*	Sensitivity (95%CI)	Specificity (95%CI)	PPV (95%CI)	NPV (95%CI)	Sensitivity (95%CI)	Specificity (95%CI)	PPV (95%CI)	NPV (95%CI)
Volume (mm^3^)	>388	75.8% [25/33] (64.8–84.4)	64.6% [31/48] (53.1–74.7)	59.5% [25/42] (48.0–70.2)	79.5% [31/39] (68.8–87.5)	42.4% [14/33] (30.7–54.9)	74.3% [26/35] (62.0–84.0)	60.9% [14/33] (48.2–72.3)	57.8% [26/45] (45.2–69.5)
JointMax_GLCM (ADC)	>0.012	69.7% [23/33] (58.3–79.3)	64.6% [31/48] (53.1–74.7)	57.5% [23/40] (46.0–68.3)	75.6% [31/41] (64.5–84.3)	60.6% [20/33] (48.0–72.1)	37.1% [13/35] (26.0–49.7)	47.6% [20/42] (35.5–60.0)	50.0% [13/26] (37.7–62.3)
GLNU_GLRLM (ADC)	>1,910	69.7% [23/33] (58.3–79.3)	68.7% [33/48] (57.4–78.4)	60.5% [23/38] (49.0–71.1)	76.7% [33/43] (65.8–85.3)	36.4% [12/33] (25.3–48.9)	77.1% [27/35] (65.0–86.3)	60.0% [12/20] (47.–71.5)	56.2% [27/48] (43.7–68.1)
RP_GLRLM (ADC)	<0.906	72.7% [24/33] (61.5–81.9)	62.5% [30/48] (51.0–72.9)	57.1% [24/42] (45.7–68.0)	76.9% [30/39] (66.0–85.4)	63.6% [21/33] (51.01–74.8)	68.6% [24/35] (56.0–79.1)	65.6% [21/32] (53.0–76.5)	66.7% [24/36] (54.2–77.5)

Numbers in square brackets represent the number correctly classified over the total number of each class.

PPV, positive predictive value; NPV, negative predictive value; ADC, apparent diffusion coefficient.

*Determined by Youden’s index considering the high aggressive tumor as the positive class.

### 3.2 Multiparametric Analysis

#### 3.2.1 Feature Selection

**Figure 4:**
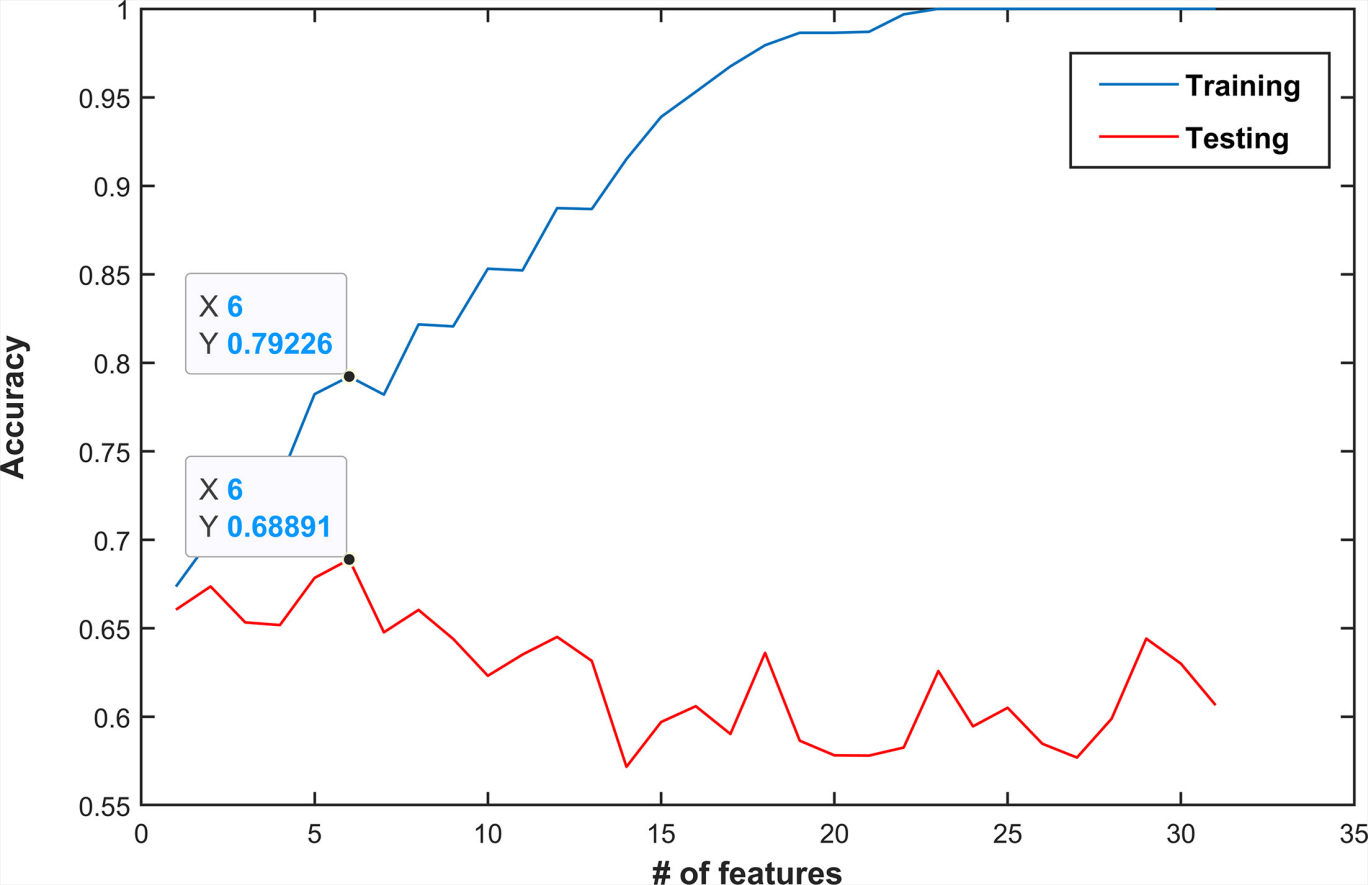
Classification performance (mean accuracy of a k-fold validation) as a function of the number of features used. Features are ordered by decreasing area under the receiver operating characteristic curve (AUC) values. In this example, the best performance was obtained when the first six features were used (mean accuracy of 0.79 and 0.69 in the training and test set, respectively). Choosing more features would lead to overfitting and thus decrease performance.

**Figure 4** shows the mean accuracy of the k training sets and test sets obtained by each of the n SVM classifier, i.e., trained with the nth subset. Overfitting, i.e., the point where performance on a training set keeps increasing, while that on the test set starts decreasing, occurs after the sixth subset, i.e., containing the six best and not correlated features ranked by their AUC values. The best feature subset is composed of six features: volume, two features derived from the T2w image (difference_average _GLCM and RP_GLRLM), and three features computed on the ADC maps (JointMax_GLCM and RP_GLRLM). Three out of four features with AUC ≥ 0.7 were kept in the best feature subset, while GLNU_GLRLM from ADC was discarded since it was highly correlated with lesion volume (ρ = 0.996). The parameter C of the SVM was set to 4, after having performed a grid search using the same k-folds used to select the best feature subset.

#### 3.2.2 Classification Performances

**Figure 5:**
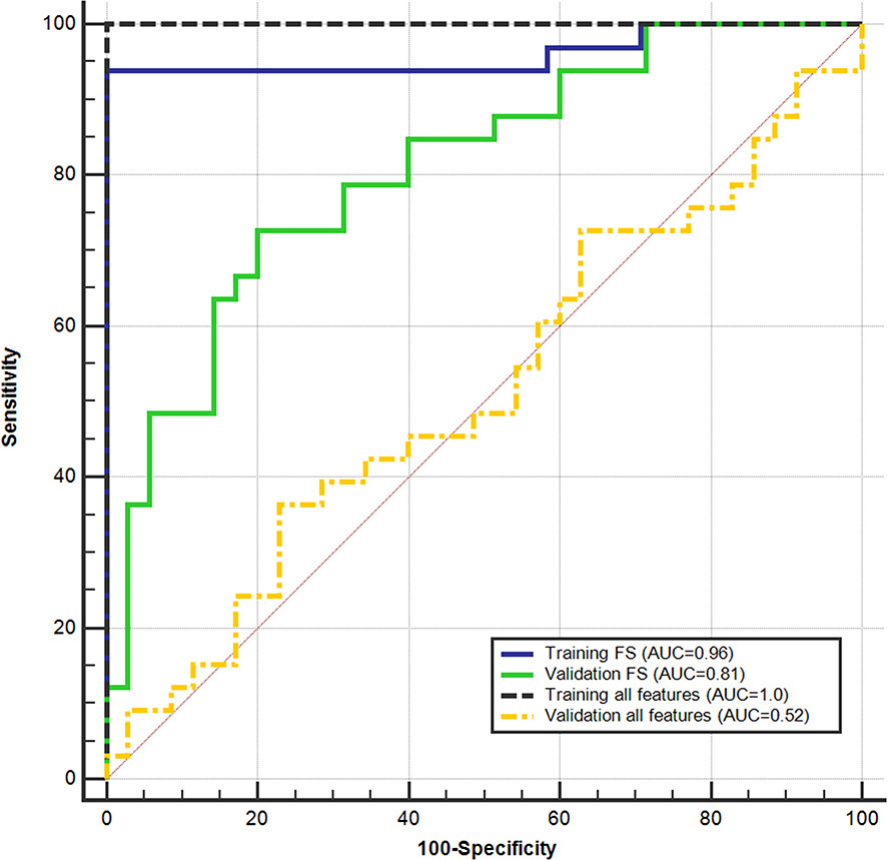
Support vector machine (SVM) performance on the training set and validation set in discriminating between low (GG ≤ 2) and high (GG > 2) aggressive prostate cancers. receiver operating characteristic (ROC) curves were obtained using all parameters and after applying the feature selection (FS).

**Figure 5** shows the ROC curves achieved for the discrimination of GG ≤ 2 PCas and GG > 2 PCas on the training and validation sets, with and without FS. With the use of all features, the classifier obtained perfect results on the training set, yet not statistically different than those obtained using the chosen feature subset. However, on the validation set, performances plunged and were statistically lower than those obtained after applying FS. This behavior clearly showed that overfitting occurs when applying a high number of features.

Results obtained on the training set at the cost-effective cutoff (0.42), after applying FS, are shown in **Table 3**. Accuracy was 95.1% (77/81; 95%CI: 87.8–98.9) and 75.0% (51/68; 95%CI: 63.0–84.7) on the training and validation sets, respectively. The cutoff that maximizes NPV computed on the training set was 0.39, and the results are shown in **Table 3**. Performances were not statistically different between the validation sets of the two centers.

**Table 3:** Performance of multiparametric analysis on both training and validation sets.

		Training set				Validation set			
Feature	Best thresh*	Sensitivity (95%CI)	Specificity (95%CI)	PPV (95%CI)	NPV (95%CI)	Sensitivity (95%CI)	Specificity (95%CI)	PPV (95%CI)	NPV (95%CI)
Youden’s index	>0.42	93.9% [31/33] (79.9–99.3)	95.8% [46/48] (85.7–99.5)	93.9% [31/33] (79.9–99.3)	95.8% [46/48] (85.7–99.5)	63.6% [21/33] (45.1–79.6)	85.7% [30/35] (69.7–95.2)	80.8% [21/26] (64.2–90.8)	71.4% [30/42] (60.9–80.0)
Cutoff that maximizes NPV	>0.39	100% [33/33] (89.4–100)	22.9% [11/48] (12.0–37.3)	43.3% [33/70] (47.1–51.0)	100% [11/11] (71.5–100)	84.8 [28/33] (68.1–94.9)	57.2% [20/35] (39.4–73.7)	65.1% [15/43] (55.4–73.7)	80% [20/25]; (62.9–90.4)

Numbers in square brackets represent the number correctly classified over the total number of each class.

PPV, positive predictive value; NPV, negative predictive value.

*Determined by Youden’s index considering the high aggressive tumor as the positive class.

**Figure 6:**
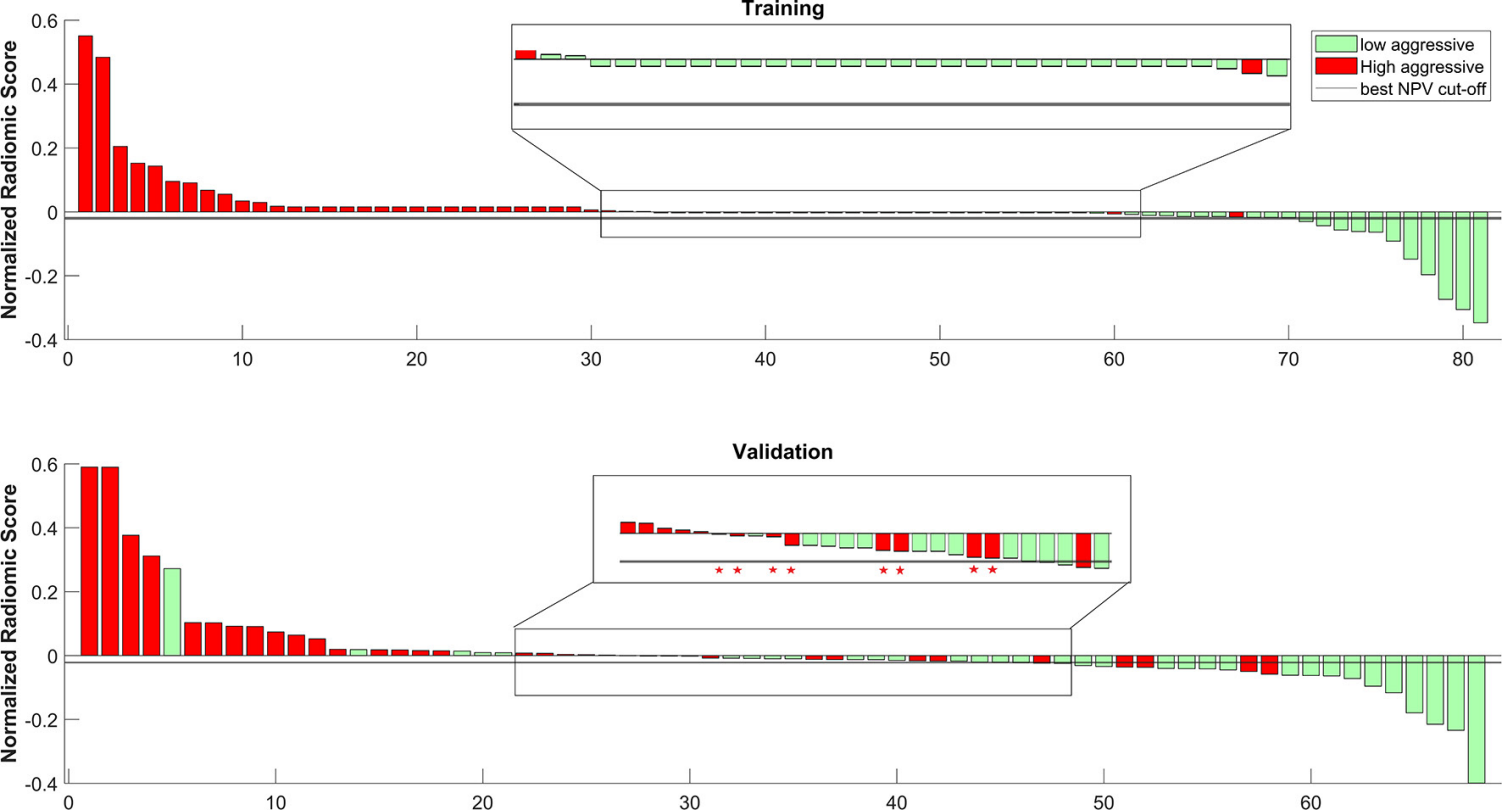
Waterfall plot of both training and validation sets. The black line shows the cutoff that maximizes the negative predictive value (NPV). * shows the eight tumors that were correctly classified as high aggressive when using the cutoff that maximizes NPV.

**Figure 7:**
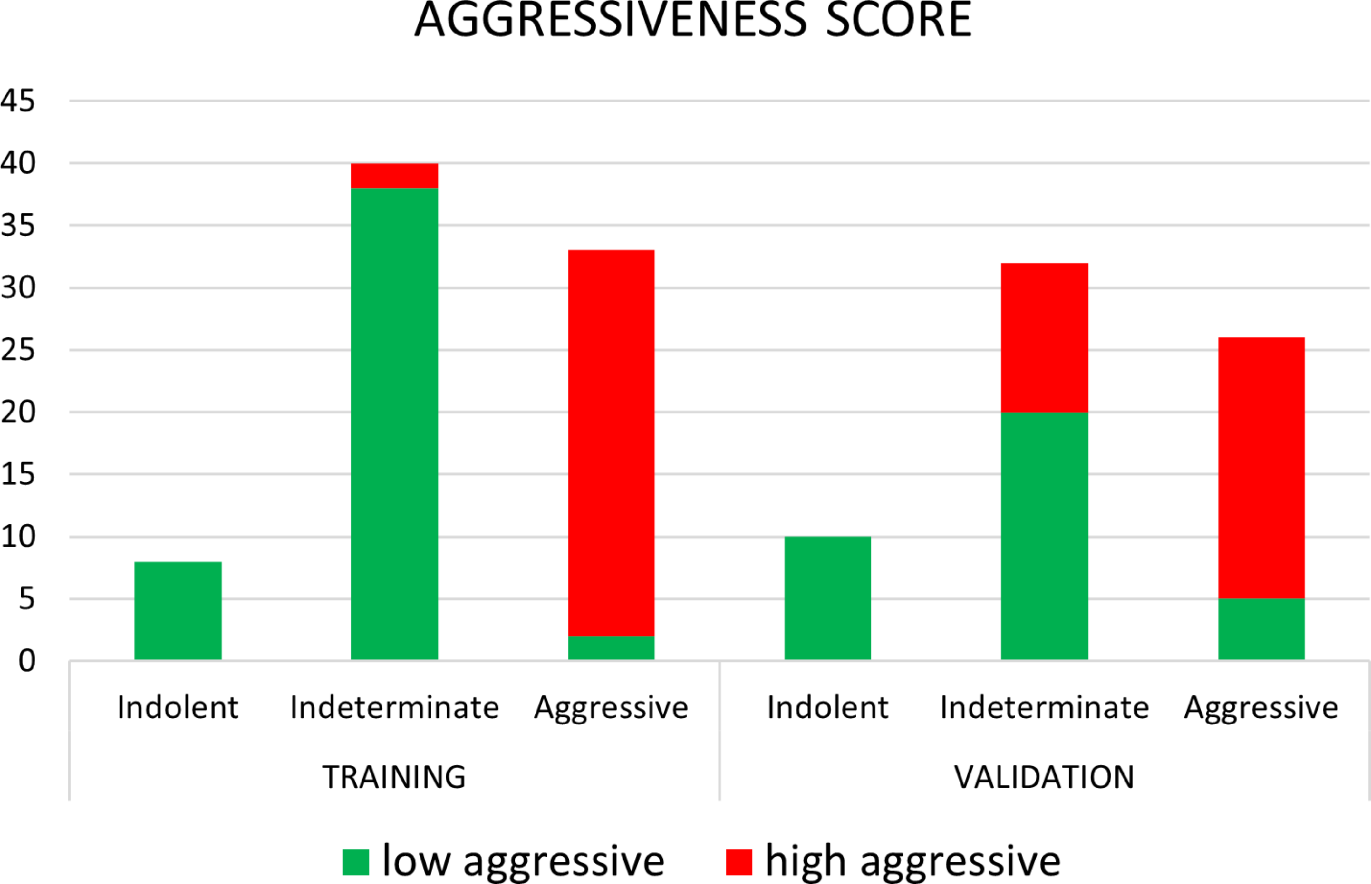
Distribution of low aggressive and high aggressive tumors in the three levels of aggressiveness score, for both the training and validation sets.

**Figure 8:**
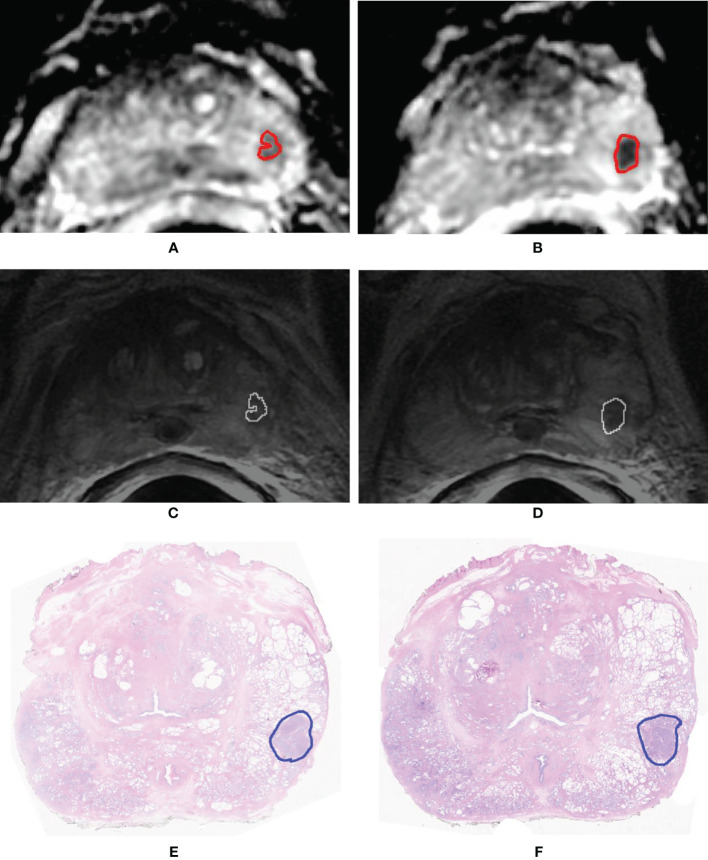
Example of two slices of a GS = 4 + 4 (GG 4) prostate cancer having volume = 0.84 cm^3^ that was erroneously classified as low aggressive. Segmentation mask superimposed to the apparent diffusion coefficient (ADC) map **(A, B)** and to the T2-weighted (T2w) image **(C, D)**. Histopathologic section of the tumor in which the pathologist drew the outline of the tumor (blue contour) **(E, F)**.

**Figure 6** shows the waterfall plot of the radiomics score of the classifier on both the training and validation sets. The black line shows the position of the cutoff that maximizes NPV. When this threshold is chosen, all aggressive tumors in the training set are correctly classified; i.e., there is no red bar with negative normalized radiomics score. Moreover, in the validation set, eight high aggressive tumors that were erroneously classified as low aggressive with the best cutoff were correctly classified, when using this cutoff, i.e., the eight red bars with a negative normalized radiomics score (**Figure 6**). **Figure 7** shows the distribution of low and high aggressive tumors within the three levels of the Agg-score. All FNs in both the training (2/2) and validation sets (12/12) were classified as indeterminate, meaning that further investigations are needed, while no high aggressive tumor was classified as indolent (not likely to be aggressive), and only 2/33 and 5/26 low aggressive tumors were classified as high aggressive, in the training and validation sets, respectively. Therefore, our method provided a clear indication of aggressiveness (Agg-score indolent and aggressive) in 51% and 53% of lesions in the training and validation sets, respectively, without classifying as non-aggressive any high aggressive tumor. Two examples of misclassified lesions are shown in **Figure 8**.

## 4 Discussion

In this study, we present a comprehensive non-invasive tool that can detect PCa and provide a likelihood score of cancer aggressiveness. Our system provides a radiomics score based on texture features extracted from the automatic segmentation of the tumor, reaching a considerable accuracy in discriminating between low and high aggressive PCa (AUC respectively of 0.96 and 0.81 in the training and validation datasets).

The fully automatic pipeline we developed represents an important improvement with respect to previous studies, which relied mainly on manual segmentations performed by expert radiologists. Manual segmentation is a time-consuming task and is impractical when large databases need to be evaluated (49). Moreover, results are operator dependent and not readily transferrable into clinical practice (30, 50). An additional advantage of our CAD system is that it has been designed with the purpose of effectively managing patients with PCa, also allowing the distinction of PCa in three groups, i.e., aggressive, indolent and indeterminate. Each group leads to a different diagnostic/therapeutic path that will be customized to the actual clinical need of each patient. Indeed, if further validation will confirm our findings, men classified with aggressive PCa could undergo radical treatments, while conversely, those with indolent tumors could benefit from active surveillance. Both could avoid the side effects of multiple core biopsies, paving the way to a new diagnostic paradigm where tissue biopsy is substituted by virtual biopsy. Finally, in patients with indeterminate findings, additional information will be needed for decision making, for example, by retrieving additional biopsy samples of the lesion or considering other clinical, laboratory, and molecular variables. To further improve the accuracy of the diagnostic assessment of patients with PCa, in the future, we envisage the integration of our PCa aggressiveness score into a more comprehensive clinical decision support system.

Other studies have assessed the relationship between quantitative biomarkers and PCa aggressiveness. Of note, Rozenberg et al. (23) developed an SVM classifier that used four texture parameters derived from ADC maps, i.e., skewness, kurtosis, entropy, and run length non-uniformity. On a dataset of 54 patients, all GS = 7 tumors, they obtained an AUC of 0.77, a sensitivity of 71%, and a specificity of 78% in distinguishing between GS ≤ 3 + 4 and GS > 3 + 4 at the best cutoff point, using a 10-fold CV method. In comparison, our study yielded higher AUC values. Moreover, the present study included tumors from GS = 6 to GS ≥ 8, increasing heterogeneity of the dataset and providing a setting more similar to the actual clinical practice. Previously, ADC-derived biomarkers have been also used to predict PCa aggressiveness. For example, Wibmer et al. (25) demonstrated that ADC energy is lower and ADC entropy is higher in tumor with GS > 3 + 4, and Rosenkrantz et al. (22) observed that ADC entropy values were higher in tumors with a GS = 4 + 3 than in tumors with a GS = 3 + 4. Finally, Nketiah et al. (26) demonstrated that energy and entropy derived from T2w images were moderately correlated with GS. As a general consideration, all of the above-reported studies did not evaluate the combination of different parameters and if a combined radiomics score could increase performances in assessing PCa aggressiveness.

More recently, some authors did explore ML to distinguishing GS = 6 PCa from more aggressive tumors by using CV, with promising results (49, 51–54). However, among these authors, only Varghese et al. validated their algorithms on an independent cohort, obtaining an AUC of 0.71 in distinguishing between GS = 6 and higher (53).

From a pathological perspective, it has now been cleared that the new GG correlates with PCa-specific mortality (10). In particular, GG groups 1 and 2 have a more favorable prognosis with respect to GG 3 through 5. Our CAD system was specifically designed to distinguish these two cohorts of patients. Previously, only Chaddad et al. (51) provided an analysis of the usefulness of radiomics features in distinguishing between patients with a favorable outcome from those with an unfavorable prognosis, i.e., GS ≤ 3 + 4 or GG 1 and 2 *versus* GS > 3 + 4 or GG 3 through 5. However, they obtained an AUC of 0.64 on an independent dataset of 20 patients from the same institution, while our method reached an AUC of 0.81 on an independent cohort composed of images acquired from two different institutions, which is important for ensuring robustness and generalizability of the tool.

Another important strength of our study relies on the fact that we demonstrated that integrating 3D texture features from both T2w images and ADC maps into an ML algorithm could provide a more precise classification of men with PCa between low and high aggressive cases than those provided by using only the ADC map (AUC = 0.96 *vs.* AUC = 0.55, respectively). We are aware that computing ADC value from a manually drawn ROI might appear more easy to do and practical than running ML from a scratch. However, although the ML-based classifier derivation process may seem involved, the clinical practitioners do not have to deal with it directly. The resultant classifier, which can typically be implemented in a few lines of code on top of the existing radiomics pipeline, can be executed by such practitioners through a simple graphical user interface (GUI) and only a few clicks of a mouse or key (53).

Our study has also some limitations. First, we did not include transitional zone tumors in the analysis. However, tumors in the transitional zone have different texture characteristics from those in the peripheral zone (55), requiring a different computational approach. Second, our assessment of aggressiveness was limited to the analysis of two large classes of tumors, i.e., those with a favorable *versus* those with a poor prognosis. For example, we did not attempt a correlation between radiomics score and percentage of GG 4, which has important prognostic implications (56). Finally, we are aware that a more extensive validation process will be necessary on different MRI equipment and using different protocols, to confirm the strength and generalizability of our findings.

In conclusion, in this study, we developed a CAD system, based on radiomics features, that automatically segments PCa, providing an aggressiveness likelihood map capable of distinguishing tumors with a favorable outcome from those with a poor prognosis. Our CAD performance is superior to that of previous studies and overcomes some of their limitations, such as the need to perform manual segmentation or the fact that analysis is limited to single-center datasets. The results of this study are promising and could pave the way to a prediction tool for personalized decision making in patients harboring PCa.

## Data Availability Statement

The raw data supporting the conclusions of this article will be made available by the authors, without undue reservation.

## Ethics Statement

The studies involving human participants were reviewed and approved by Comitato etico Azienda Ospedaliera San Luigi Gonzaga and by Comitato etico della Fondazione del Piemonte per l’Oncologia, Istituto di Candiolo. The patients/participants provided their written informed consent to participate in this study.

## Author Contributions

VG, SM, and DR contributed to the conception and design of the study. GS, MC, EB, MB, FP, MM, AP, AV, and FR participated in the collection, curation, and report of data. VG developed the algorithms. VG, SM, and AD performed the statistical analysis. VG wrote the first draft of the manuscript. SM and DR wrote sections of the manuscript. All authors contributed to the article and approved the submitted version.

## Funding

The research leading to these results has received funding from the Fondazione AIRC under IG2017 - ID. 20398 project – P.I. Daniele Regge, from the European Union’s Horizon 2020 research and innovation program under grant agreement no. 952159 and from Fondazione Cassa di Risparmio di Cuneo, Bando Ricerca Scientifica 2015-2016, ID. 2016-0707.

## Conflict of Interest

The authors declare that the research was conducted in the absence of any commercial or financial relationships that could be construed as a potential conflict of interest.

## Publisher’s Note

All claims expressed in this article are solely those of the authors and do not necessarily represent those of their affiliated organizations, or those of the publisher, the editors and the reviewers. Any product that may be evaluated in this article, or claim that may be made by its manufacturer, is not guaranteed or endorsed by the publisher.
