# Supplementary material for: A Fully Automatic Artificial Intelligence System Able to Detect and Characterize Prostate Cancer Using Multiparametric MRI: Multicenter and Multi-Scanner Validation
Source: Front Oncol. 2021 Oct 1;11:718155. doi: 10.3389/fonc.2021.718155 (PMC8517452; doi:10.3389/fonc.2021.718155)
Supplement: Supplementary file 1 [file Table_1.docx]

Supplementary Table 1: MRI sequence details for the different types of acquisitions

|  | **Sequence** | | **spacing** | **ST (mm)** | **FOV (cm)** | **NEX** | **AM** | **RM** | **TR/TE/FA** | **Additional**  **information** |
| --- | --- | --- | --- | --- | --- | --- | --- | --- | --- | --- |
| **CENTER A** | T2w (fast spin echo - FSE) | | 0.3168x0.3168 | 3 | 16x16 | 18 | 384x288 | 512x512 | 3020/85/  160° |  |
|  | DW (EPI)* | | 0.625x0.625 | 3 | 16x16 | 1 | 128x128 | 256x256 | 7000/101/  90° | b: 0-1000 s/mm^2^ |
|  | DW (single shot EPI - FOCUS)** | | 0.625x0.625 | 3 | 16x16 | 1 | 140x70 | 256x256 | 3200/73/  90° | b: 0-1000 s/mm^2^  %FOV = 50 |
|  | DCE (3D SPGR echo ) | | 0.3906x0.3906 | 3 | 20x20 | 1 | 224x192 | 512x512 | 3.6/1.3/  20° | temporal resolution= 13 s 26 time points |
| **CENTER B** | T2 (spin echo - SE) | | 0.556x0.556 | 3 | 16x16 | 16 | 268x224 | 288x288 | 5112/110/  90° |  |
|  | DWI | | 1.25x1.25 | 3 | 28x28 | 59 | 112x110 | 224x224 | 4790/84/  90° | b: 0-1000 s/mm2 |
|  | DCE | | 0.972x0.972 | 3 | 28x28 | 50 | 188x187 | 288x288 | 4.27/2.01/  12 | Temporal resolution= 11 s 26 time points |
|  |  | ST = slice thickness; FOV = field of view; NEX = number of excitations; NEX = number of excitations; AM = acquisition matrix; RM = reconstruction matrix; TR/TE/FA = repetition time/echo time/flip angle; * for acquisitions between April 2010 and November 2012; ** for acquisitions between November 2014 and September 2016. | | | | | | | | |

Supplementary table 2: Reporting Guidelines: from Image processing to features calculation steps.

| **Area** | **Topic** | **Description** |
| --- | --- | --- |
| **Patient** | Region of interest | Prostate |
|  | Contrast agent (only for lesion detection) | Power injection of 0.1 mmol/kg gadobutrol (Gadovist, Bayer Schering, Berlin, Germany) through a peripheral line at 0.7 ml/s, followed by infusion of 20 cc normal saline at same rate. |
| **Acquisition** | Acquisition protocol | Different acquisition protocols described in the text |
|  | Scanner type | Signa Excite HD (GE Healthcare), Optima MR450w (GE Healthcare), Achieva (Philips) |
|  | Imaging modality | MR |
|  | Scan duration | Approximately 40 minutes |
| **Image registration** | Registration method | Rigid registration to align DCE and T2w images and elastic registration to align DWI and T2w images (described in doi:10.1166/jmihi.2015.1518). |
| **Data conversion** | ADC computation | ADC= ln(S_0_/S_1_)/(b_1_-b_0_), where S_0_ and S_1_ are the signal intensity obtained with the b_0_ and b_1_ b values, respectively. |
|  | Other data conversions | Quantitative parameters from DCE images are derived from phenomenological universalities (PUN) formalism (doi: 10.1088/0031-9155/56/3/004). |
| **Post-acquisition processing** | Anti-aliasing | Recursive gaussian filter (σ=0.5 mm) |
|  | Non-uniformity correction | N4 bias field correction |
|  | Intensity normalization | T2w images are normalized using the value of the obturator muscle |
| **Segmentation** | Method | Automatic segmentation (doi: 10.1016/j.compmedimag.2015.09.001) performed using normalized T2w images, ADC maps and quantitative parameters from DCE sequences |
|  | Conversion to mask | NIFTI |
| **Image Interpolation** | Interpolation method | Trilinear interpolation performed aligning grid centers (rounded to the nearest integer). |
|  | Voxel dimension | 0.5 mm |
| **Image Interpolation** | Interpolation method | Nearest integer interpolation performed aligning grid centers (rounded to the nearest integer). |
|  | Partially masked voxel | Threshold of 0.5 |
| **Re-segmentation** | Method | Between 1^st^ and 99^th^ percentile |
| **Discretization** | Method | Fixed bin number (32 bin) |
| **Image transformation** | Image filter | NONE |
| **Image biomarker computation** | Biomarker set | Intensity-based statistics (from ADC maps), intensity histogram (from ADC maps), GLCM and GLRLM (from ADC maps and T2w images) |
|  | IBSI compliance | Yes |
|  | Software availability | C++ in-house algorithm |
| **Image biomarker computation - texture parameters** | Texture matrix aggregation | 3D merged |
|  | Distance weighting | No weighting |
|  | CM symmetry | Symmetric co-occurrence matrices |
|  | CM distance | 1 |
| **List of features** | Intensity-based (ADC only) | mean, 25th, 50th, 75th percentiles, skewness, kurtosis, intensity kurtosis and intensity variance |
|  | Intensity histogram (ADC only) | mean |
|  | GLCM (T2w and ADC) | Joint Max,  Joint Average,  Joint Variance,  Joint Entropy,  Difference Average,  Difference Variance,  Difference Entropy,  Sum Average,  Sum Variance,  Sum Entropy,  Angular Second Moment,  Contrast,  Dissimilarity,  Inverse Difference,  Normalised Inverse Difference,  Inverse Difference Moment,  Normalised Inverse Difference Moment,  Inverse Variance,  Correlation,  Autocorrelation,  Cluster tendency,  Cluster shade  Cluster prominence,  Information Correlation 1,  Information Correlation 2. |
|  | GLRLM (T2w and ADC) | Short runs emphasis,  Long runs emphasis,  Low grey level run emphasis,  High grey level run emphasis,  Short run low grey level emphasis,  Short run high grey level emphasis,  Long run low grey level emphasis,  Long run high grey level emphasis,  Grey level non-uniformity,  Normalised grey level non-uniformity,  Run length non-uniformity,  Normalised run length non-uniformity,  Run Percentage,  Grey level Variance,  Run length Variance,  Run Entropy. |
